# Outpatient prescription practices in rural township health centers in Sichuan Province, China

**DOI:** 10.1186/1472-6963-12-324

**Published:** 2012-09-18

**Authors:** Qian Jiang, Bo Nancy Yu, Guiying Ying, Jiaqiang Liao, Huaping Gan, James Blanchard, Juying Zhang

**Affiliations:** 1 West China School of Public Health, Sichuan University, No.17 Section 3 South Renmin Road, Chengdu, Sichuan, China; 2 Department of Community Health Sciences, Faculty of Medicine, University of Manitoba, Winnipeg, Manitoba, Canada; 3 Sichuan Health Information Center, Chengdu, Sichuan, China

## Abstract

**Background:**

Sichuan Province is an agricultural and economically developing province in western China. To understand practices of prescribing medications for outpatients in rural township health centers is important for the development of the rural medical and health services in this province and western China.

**Methods:**

This is an observational study based on data from the 4th National Health Services Survey of China. A total of 3,059 prescriptions from 30 township health centers in Sichuan Province were collected and analyzed. Seven indicators were employed in the analyses to characterize the prescription practices. They are disease distribution, average cost per encounter, number of medications per encounter, percentage of encounters with antibiotics, percentage of encounters with glucocorticoids, percentage of encounters with combined glucocorticoids and antibiotics, and percentage of encounters with injections.

**Results:**

The average medication cost per encounter was 16.30 Yuan ($2.59). About 60% of the prescriptions contained Chinese patent medicine (CPM), and almost all prescriptions (98.07%) contained western medicine. 85.18% of the prescriptions contained antibiotics, of which, 24.98% contained two or more types of antibiotics; the percentage of prescriptions with glucocorticoids was 19.99%; the percentage of prescriptions with both glucocorticoids and antibiotics was 16.67%; 51.40% of the prescriptions included injections, of which, 39.90% included two or more injections.

**Conclusions:**

The findings from this study demonstrated irrational medication uses of antibiotics, glucocorticoids and injections prescribed for outpatients in the rural township health centers in Sichuan Province. The reasons for irrational medication uses are not only solely due to the pursuit of maximizing benefits in the township health centers, but also more likely attributable to the lack of medical knowledge of rational medication uses among rural doctors and the lack of medical devices for disease diagnosis in those township health centers. The policy implication from this study is to enhance professional training in rational medication uses for rural doctors, improve hardware facilities for township health centers, promote health education to rural residents and establish a public reporting system to monitor prescription practices in rural township health centers, etc.

## Background

With rapid development and reform of the health services system, the focus of medication utilization in China has gradually changed from improving the accessibility of medications to the rational use of medications, namely, to use medications safely, effectively and economically [1]. Prescribing medication rationally is playing a key role in reducing the health care burden and improving health conditions of the general population in China.

Since the founding of the People’s Republic of China in 1949, public health prevention and health care services have been organized around a three-tier health care delivery system. In urban areas, the three-tier network is composed of community health service centers, district hospitals, and city hospitals. In rural areas, it consists of village clinics, township health centers (THCs), and county hospitals. The basic features of this system have remained the same to date [2]. Since the THCs are in the central position of the rural three-tier network, their missions are (a) to provide basic medical and public health services for the local population, (b) to provide technical training for local health workers, and (c) to provide technical guidance to the village clinics. In 2001, a new policy, titled “The Guidelines on Rural Health Sector Reform and Development” and promulgated by the central government (State Council 2001), emphasized that each township should have the ownership of one health center supported by the local government. This policy gives the THCs government-owned status with responsibility of providing basic health services for hundreds of millions of rural residents. The THCs play an important role in the national health services system for controlling infectious diseases, improving the quality of health services and guaranteeing people’s health in rural areas of China, and were regarded as one of the “three magic weapons (Rural Cooperative Medical Schemes, barefoot doctors and township health centers)” of the rural health services of China by WHO [3].

The 4th National Health Services Survey (NHSS, 2008) was conducted when a new round of medical and health system reform was launched. The main purpose of this survey is to provide baseline information for the implementation and assessment of the new round of medical and health system reform of the past five years, and to provide evidence for the "Healthy China 2020" plan in terms of objectives, targets and major actions [4]. Understanding the outpatient medication prescription practice in the primary health care institutions is important to guide the rational use of medications, to reduce the cost of the prescriptions, and to support evidence-based health care reform and policy development for both local and central governments. Therefore, the Ministry of Health of China (MoH) conducted the Outpatient Prescription Survey, as an additional part of the 4th NHSS in 2008.

Since China launched the strategy of “Developing the Western Region” in 2000 (covering 380 million people in 12 provinces), the central government has given vigorous supports to the development of medical and health service in the economically depressed western region. Up to 2010, the central government had invested 78.62 billion Yuan ($12.48 billion, 1 USD = 6.30 RMB Yuan) in total to 12 western provinces, which accounted for 43.3% of the whole national medical service budget of 181.40 billion Yuan ($28.79 billion) [5]. That means, the health expenditures per capita devoted by the central government in western China was about 206.9 Yuan ($32.8) in the past 10 years. Sichuan is a typical agricultural province with a large population in the western region. By the end of 2008, there were 50.944 million rural residents, 181 counties (or cities or districts), 4544 towns and 4803 THCs in Sichuan. Therefore, it is of great importance to investigate the medical prescription practice in the THCs of this province to inform the development and reform of the rural health service system in western China.

The aims of this study are to (a) describe outpatient prescription practices in the rural THCs in Sichuan Province, (b) assess any irrational malpractice in medical prescriptions and (c) explore the possible reasons and provide some suggestions for solving the problems in rural medical prescription practice.

## Methods

### Data source

The data used in this study came from the Outpatient Prescription Survey of the THCs in Sichuan Province, which was a part of the 4th National Health Services Survey (NHSS) of China in 2008.

Using a three-stage probability proportion to size sampling method, the NHSS randomly selected 30 counties in Sichuan Province in the first stage, one town from each sampled counties in the second stage, and one THC from each sampled town in the third stage for the Prescription Survey. To sample outpatient prescriptions, the systematic random sampling method was used to select 25 outpatient prescriptions on 17th September and 18th December of 2007, and 19th March and 20th June of 2008, respectively. If the total number of the prescriptions was less than 25 on a sampling day, the sampling would continue on the next day and so on until the number of prescriptions reached 25. Excluded from the sample unit were the prescriptions of emergency, infection, surgery and traditional Chinese medicine decoction pieces (prepared Chinese herbal medicine in small pieces ready for decoction, and medicine materials in crude slices), as well as laboratory test reports.

The 4th NHSS was designed by an expert panel from the Center for Statistics Information, MoH of China, and was composed of four sub-surveys: the Household Health Survey, the Health Institution Survey, the Prescription Survey and the Medical Staff Survey. All four sub-surveys were implemented in the same city/town/district at the same time. The three-stage probability proportion to size sampling method was adopted to ensure that about 1% of the population in each sampling province be included in the Household Health Survey, and that a sufficient sample size be obtained in the Health Institution Survey and the Prescription Survey. Prior to the formal survey, a pilot study was conducted by the MoH of China. Results of the pilot study showed that the average number of daily outpatient prescriptions among the THCs was about 25 per day. Statistical experts from the Center for Statistics Information estimated that 100 prescriptions sampled from each THC would be sufficient to represent the basic annual prescription practice of the THC. The selected four days in the Prescription Survey represented the four seasons: spring, summer, autumn and winter respectively. These dates also represented one day of a week, excluding weekends, statutory holidays and other special days. In short, this sampling method was aimed at adequate sample size and the representativeness of the sample.

Questionnaires of the Prescription Survey were designed by the expert panel from the Center for Statistics Information, MoH. The county or district or city level health bureaus were responsible for administrating survey training, data collection and quality checking of the questionnaires. It was mandatory that each sampled county (or district or city) set up a Quality Assessment Team to check the quality of the survey in the data collection process, and that 5% of the questionnaires was randomly selected for review after the completion of the survey. In addition, the MoH assigned a group of experts to supervise the survey. The Center for Statistical Information developed the software for data entry and management. Double data entry and validation procedures were adopted to minimize human errors in data entry. The data checking that occurred in each sampled county (or district or city) was strictly monitored by the designated persons in the provinces.

Since the NHSS is a national survey organized by the MoH, all health institutions and individuals involved had responsibilities and obligations to cooperate with the investigators. The 4th NHSS was approved by the ethics committees of the Center for Statistics Information, MoH. In addition, the Prescription Survey was completely anonymous, and fully took human rights and ethical issues into consideration when it was designed, so the prescribers would not care whether they were being monitored at all. The Contract units, such as Sichuan Health Information Centre and Sichuan University, were only responsible for data collection and analysis.

### Analysis

Contents of the Prescription Survey included: distribution of different disease diagnoses, costs of prescriptions, and utilization practices (numbers and types) of different medications. Seven indicators were used for description in this study: (1) Distributions of the top 10 common diseases; (2) Average cost per encounter; (3) Average number of medications per encounter; (4) Percentage of encounters with antibiotics prescribed; (5) Percentage of encounters with glucocorticoids prescribed; (6) Percentage of encounters with combined glucocorticoids and antibiotics prescribed; (7) Percentage of encounters with injections prescribed. Among the above indicators, (2), (3), (4), (5) and (7) are WHO standard indicators [6].

The definitions of ‘antibiotic’, ‘glucocorticoid’ and ‘injection’ in this article are consistent with the standard definitions of the 4th NHSS. ‘Antibiotic’ refers to synthetic antibacterial medications and antimicrobial agents of non-plant components including penicillin and medications of other anti-bacterial, anti-skin infection, anti-ophthalmic infection and anti-diarrhoeal that contain gentamicin, quinolones or other compounds. Medications of anti-filariasis, anti-leprosy, anti-TB, anti-fungal and anti-malarial were not defined as ‘antibiotics’. ‘Glucocorticoid’ refers to systemic glucocorticoids, excluding local glucocorticoids, such as those applied topically. ‘Injection’ is exclusive of vaccination, solvent, systemic or local anesthesia, or subconjunctival and retrobulbar injection.

## Results

Final data available for the analysis in this study consist of 3059 prescriptions from 30 rural THCs in Sichuan Province.

### Distributions of diseases

In Table 1, we list the top 10 common diseases according to the frequency of their prescriptions. It is clearly shown that the majority of diagnoses were in the categories of respiratory and digestive system diseases. Upper respiratory tract infection (URTI), gastroenteritis, common cold, chronic obstructive pulmonary disease (COPD) and female genital disease were at the top five diagnoses. Hypertension was ranked tenth among the most common prescribed diseases.

**Table 1:** Proportion and average cost of the top 10 common disease prescriptions

**Rank**	**Diagnoses**	**Number (proportion)**	**Average cost (median, RMB**^**a**^**)**	**Average cost (median, USD)**	**Range of cost (RMB**^**a**^**)**	**Range of cost (USD)**
1	Upper respiratory tract infection	652 (21.31%)	13.00	2.06	0.40 - 147.00	0.06 - 23.33
2	Gastroenteritis	344 (11.25%)	16.35	2.59	0.60 - 204.40	0.10 - 32.44
3	The common cold	272 (8.89%)	10.00	1.59	0.80 - 119.00	0.13 - 18.89
4	COPD	198 (6.47%)	18.90	3.00	1.20 - 184.00	0.19 - 29.20
5	Female genital diseases	148 (4.84%)	30.55	4.85	1.00 - 138.40	0.16 - 21.97
6	Pneumonia	86 (2.81%)	31.50	5.00	2.50 - 194.30	0.40 - 30.84
7	Rheumatoid arthritis	80 (2.62%)	17.40	2.76	1.70 - 156.00	0.27 - 24.76
8	Other digestive tract diseases	73 (2.39%)	10.00	1.59	1.00 - 106.15	0.16 - 16.85
9	Dental diseases	63 (2.06%)	14.00	2.22	1.00 - 144.00	0.16 - 22.85
10	Hypertension	61 (1.99%)	20.70	3.29	4.00 - 215.30	0.63 - 34.17

^a^1 USD = 6.3009 RMB Yuan.

### Average cost per prescription

The median cost per encounter was 16.30 Yuan ($2.59). Among the prescriptions for the top 10 common diseases, the highest median cost was for pneumonia and female genital diseases, 31.50 Yuan ($5.00) and 30.55 Yuan ($4.85), respectively as shown in Table 1.

### Utilization of Chinese patent medicine

Chinese patent medicine (CPM) also known as Zhongcheng Yao in Chinese. "Patent" refers to the standardization of the formula. Specifically, CPM uses traditional medicine and herb drugs as raw materials and processes those raw materials into certain dosage forms according to the prescription book and refining method. Patents may come in the forms such as teapills, dripping pills, liquids, syrups, powders, granules, instant teas, and capsules. In China, many of CPMs have been well-known and widely used, due to their features of curative effect, convenience and inexpensiveness. They are often used when a patient's condition is not severe or urgent and the medicine can be taken as a long-term treatment. So the utilization of CPM was included in the Prescription Survey of the 4th NHSS.

Close to 60% of the prescriptions contained CPM, and the median number of CPM per prescription was one. Approximately 20% of the prescriptions contained two or more CPMs (see Table 2). 94.00% of the prescriptions for hypertension contained CPM, which was the highest among the top 10 common diseases (see Table 3).

**Table 2:** Proportion of prescriptions containing CPM and western medicine (%)

**Type of the medications**	**Number of medications per encounter**				**Total**
	**0**	**1-3**	**4-5**	**≥6**	
Chinese patent medicine	43.84	55.54	0.52	0.10	100.00
Western medicine	1.93	36.64	43.42	18.01	100.00
Total	0.29	21.74	46.68	31.29	100.00

**Table 3:** Proportion of prescriptions with CPM and western medicine of the top 10 common diseases (%)

**Rank**	**Diagnoses**	**Number of Chinese patent medicine**						**Number of western medicine**						
		**0**	**1**	**2**	**3**	**4**	**≥5**	**0**	**1**	**2**	**3**	**4**	**5**	**≥6**
1	Upper respiratory tract infection	23.34	45.35	26.00	4.74	0.19	0.38	0.15	3.24	8.47	24.81	25.73	21.11	16.49
2	Gastroenteritis	32.43	45.56	19.31	1.93	0.39	0.39	0.00	2.92	7.02	23.98	28.65	21.35	16.08
3	The common cold	18.64	57.27	18.64	4.55	0.00	0.91	1.18	2.76	11.81	22.05	23.62	24.02	14.57
4	COPD	20.75	53.46	23.27	2.52	0.00	0.00	0.00	2.02	6.06	18.69	25.25	22.73	25.26
5	Female genital diseases	25.00	43.52	23.15	7.41	0.93	0.00	0.68	7.53	13.70	18.49	21.23	17.81	20.55
6	Pneumonia	17.14	55.71	18.57	4.29	2.86	1.43	1.16	1.16	3.49	12.79	18.60	19.77	43.02
7	Rheumatoid arthritis	21.82	50.91	25.45	1.82	0.00	0.00	1.25	5.00	8.75	16.25	30.00	23.75	15.00
8	Other digestive tract diseases	42.59	42.59	12.96	0.00	0.00	1.85	2.74	4.11	20.55	20.55	23.29	12.33	16.44
9	Dental diseases	28.85	46.15	23.08	1.92	0.00	0.00	0.00	7.94	12.70	25.40	19.05	17.46	17.46
10	Hypertension	6.00	62.00	30.00	2.00	0.00	0.00	0.00	9.84	14.75	32.79	11.48	9.84	21.31

### Utilization of western medicine

Almost all prescriptions (98.07%) contained western medicine, and the median number of western medicines per encounter was four. 36.64% of the prescriptions contained 1–3 western medicines. Close to half (43.42%) of the prescriptions contained 4–5 western medicines. Almost one fifth (18.01%) of the prescriptions contained six or more western medicines (see Table 2). Among the top 10 common diseases, 43.02% of pneumonia prescriptions contained six or more western medicines (see Table 3).

#### Utilization of antibiotics

The majority (85.18%) of the prescriptions contained antibiotics, and the median number of antibiotics per encounter was one. A quarter (24.98%) of the prescriptions contained two or more antibiotics. In detail, prescriptions with two, three, four or more antibiotics comprised respectively 19.88%, 3.78% and 1.32% of the total. Almost all (96.86%) of the COPD prescriptions contained antibiotics, which was the highest among the top 10 common diseases, followed by pneumonia (95.12%) and female genital diseases (94.33%). About a half (48.23%) of the prescriptions for female genital diseases and 35.37% of the prescriptions for pneumonia contained two or more antibiotics. Finally, the proportions of the prescriptions with three or more antibiotics for female genital diseases, dental diseases and pneumonia were all above 8% (see Table 4).

**Table 4:** Proportion of prescriptions with antibiotics, glucocorticoids and injections of the top 10 common diseases (%)

**Rank**	**Diagnoses**	**Any antibiotics**	**Two or more antibiotics**				**Glucocorticoids**	**Glucocorticoids & antibiotics**	**Any injections**	**Two or more injections**			
			**2**	**3**	**≥4**	**total**				**2**	**3**	**≥4**	**total**
1	Upper respiratory tract infection	92.52	17.52	2.07	0.96	20.55	21.47	19.94	53.37	17.15	8.48	12.33	37.96
2	Gastroenteritis	86.20	19.63	4.60	1.84	26.07	5.28	4.94	38.78	8.98	6.53	13.06	28.57
3	The common cold	82.68	13.42	0.43	0.00	13.85	28.36	18.75	43.75	18.18	8.52	7.39	34.09
4	COPD	96.86	25.13	4.71	1.05	30.89	32.61	30.30	66.87	13.13	15.00	28.75	56.88
5	Female genital diseases	94.33	39.01	9.22	0.00	48.23	10.14	9.46	52.94	17.65	11.76	12.75	42.16
6	Pneumonia	95.12	26.83	6.10	2.44	35.37	30.86	27.91	74.24	10.61	10.61	42.42	63.64
7	Rheumatoid arthritis	72.86	12.86	4.29	0.00	17.15	42.86	25.00	32.08	5.66	3.77	11.32	20.75
8	Other digestive tract diseases	80.30	22.73	1.52	3.03	27.28	2.86	2.74	52.83	18.87	9.43	11.32	39.62
9	Dental diseases	91.94	17.74	8.06	1.61	27.41	23.73	20.63	44.68	17.02	10.64	12.77	40.43
10	Hypertension	43.75	2.08	4.17	0.00	6.25	5.00	3.28	27.91	2.33	2.33	16.28	20.94

#### Utilization of glucocorticoids

About 20% of prescriptions contained any glucocorticoids. Among the top 10 common diseases, the highest proportions of prescriptions with glucocorticoids were for rheumatoid arthritis (42.86%), COPD (32.61%) and pneumonia (30.86%), which were much higher than other diseases. Surprisingly, 28.36% of common cold (flu), 23.73% of dental disease, and 10.14% of female genital diseases were also prescribed glucocorticoids (see Table 4).

#### Utilization of combined glucocorticoids and antibiotics

In total, 16.67% of the prescriptions contained both glucocorticoids and antibiotics. Among the top 10 common diseases, the highest proportion of prescriptions combined the two was given to the diagnoses of COPD (30.30%), pneumonia (27.91%) and rheumatoid arthritis (25.00%) (see Table 4).

#### Utilization of injections

Over half (51.40%) of the prescriptions contained injections. The median number of injection prescribed per encounter was one. The prescriptions that included two or more injections consisted of 39.90% in total, with 14.19%, 9.91% and 15.80% of two, three, four or more injections respectively. Particularly, the proportion of prescriptions containing injections was 74.24% for pneumonia and 66.87% for COPD, higher than that for other diseases. Such high rates of injections used for the two diseases were also reflected by the high proportion of prescriptions that contained two or more injections, 63.64% for pneumonia and 56.88% for COPD respectively. About 40% of the prescriptions for female genital diseases, dental diseases and other digestive tract diseases contained two or more injections. Prescriptions with four or more injections were also observed among all the top 10 common diseases, with the highest three proportions of 42.42%, 28.75% and 28.75% for pneumonia, COPD and hypertension respectively (see Table 4).

### The polypharmacy practice in the THCs

The prescriptions with 1–3, 4–5 and six or more medications (western medicine or Chinese patent medicine or both) comprised respectively 21.74%, 46.68% and 31.29% of the total (see Table 2). The median number of medications per encounter was four.

**Figure 1:**
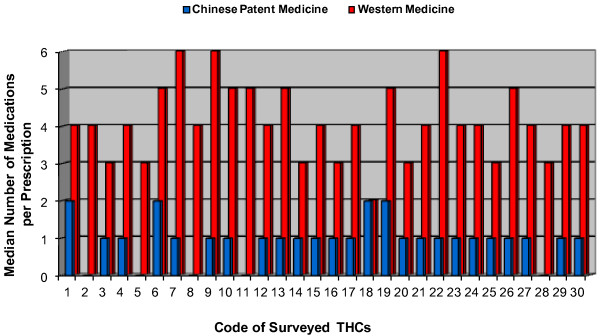
Distribution of median number of medications per prescription among the 30 THCs.

The median number of western medicines prescribed per encounter distributed differently among the surveyed 30 THCs. The median number of western medicine prescribed among the 30 surveyed THCs varied from two to six per encounter. However, this uneven distribution was not seen in the CPM prescriptions among the surveyed THCs. A greater proportion of surveyed THCs (73%, or 22 out of 30 THCs) prescribed four or more western medications per encounter versus only a few (13%, or 4 out of 30 THCs) which prescribed two or more CPMs per encounter (see Figure 1). 

## Discussion and conclusion

The WHO recommended that the average number of medications (refers to western medicines only) per encounter for outpatients should be 1.6 to 2.8 in developing countries [7]. In 2007, the Prescription Administrative Policy of China (MoH, 2007) specified that the average number of medications (refers to western medicines, or CPMs, or both) per encounter must be less than 6 [8] based on the reviewed results that when the number of medications per encounter was less than 6, 6–10, 11–15 and 16–20, the incidence of adverse reactions was 4%, 10%, 28% and 54%, respectively [9]. The average number of western medicines per encounter in the THCs in Sichuan was four, just in keeping with the national standard, but notably higher than the WHO recommended standard. However, other developing countries, such as Pakistan, also reported slightly higher number of medications (average of four per encounter) prescribed by medical practitioners [10]. Our study found that (a) more than 60% of the prescriptions did not follow the WHO recommended standard by prescribing four or more western medications; (b) more than 18% of the prescriptions did not even follow the Prescription Administrative Policy of China by prescribing six or more western medicines per encounter; and (c) the problem of over-prescribing western medications in the treatment of pneumonia was most prominent among the top 10 common diseases. Over 40% of pneumonia prescriptions had six or more western medicines.

Most clinical bacterial infections can be controlled with one antibiotic. It remains controversial whether combination antimicrobial therapy is more efficacious than monotherapy [11,12]. Nevertheless, there was wide consensus that the number of antibiotics per encounter should be controlled within two or three [13-15]. The indications for combination antimicrobial therapy only include: (a) empirical treatment of life-threatening infections; (b) treatment of polymicrobial infections; (c) prevention of the emergence of bacterial resistance; and (d) for synergism [11,16]. Otherwise, any unnecessary usage of multiple antibiotics will give rise to side effects, drug resistance, high cost and other problems [13-15]. According to a WHO report based on prescription data of 11 Asian and African countries (2002), the proportion of prescriptions with antibiotics ranged from 27% to 63%, with the noticeable low figures of 20.0%-26.8% from developing countries. However, the overall proportion of antibiotics prescription among the THCs in Sichuan was 85.18%, considerably exceeding the WHO recommendation of ‘not more than 30%’ and of the national recommendation of ‘below 50%’ proposed by the ‘Regulations on Hospital Infection Administration of China’ [17]. The proportion of prescriptions with two or more antibiotics in this study was 24.98%, much higher than the previously reported national average (15.7%) in the same year [18].

There are strict restrictions on the use of glucocorticoids. Long-term use of large doses of glucocorticoids can cause central obesity, hypertension, hyperglycemia and other adverse consequences [19,20]. The Chinese national reference level of using glucocorticoids ranged from 0% to 9.95% [1]. This study revealed a much higher level of glucocorticoids utilizations in the THCs of Sichuan, 19.99% in general, 42.86% for rheumatoid arthritis, 32.61% for COPD and 30.86% for pneumonia, and 10.14%-28.36% for other top 10 common diseases. This result suggests that the Administrative Department of Health of Sichuan provincial government should pay more attention to the unnecessary use of glucocorticoids in the THCs and develop certain policy on glucocorticoids utilization at all levels of health care providers.

A novel finding of our study was the significantly high utilization of combined antibiotics and glucocorticoids at the THCs in Sichuan Province. Some studies showed that the irrational use of combined antibiotics and glucocorticoids can lead to the spread of bacterial infection and induce other new infections [21]. This has recently become a new focus of pharmacy research in China. It has been accepted that only certain severe infections require the combined use of glucocorticoids and antibiotics. For example, the combined use of oral antibiotics and systemic corticosteroids decrease the risk of deterioration for acute exacerbations of COPD [22]. By examining the details of the combined use of antibiotics and glucocorticoids in THCs in Sichuan Province, this study revealed that there was unnecessary and irrational co-prescribing of antibiotics and glucocorticoids in the THC prescription practice. For example, even in the prescription for URTI and common cold, which did not need to take antibiotics and glucocorticoids together, yet nearly 20% of the prescriptions contained both glucocorticoids and antibiotics. We have not found any publication discussing the combinatorial usage of antibiotics and glucocorticoids with exact proportions in western China before.

Injections are the most frequent medical procedure performed throughout the world. About 16 billion injections are administered each year in developing and transitional countries [23]. Overuse of injection not only increases the health care costs, but also increases potential spread of iatrogenic diseases and affects the quality of clinical care [24,25]. According to the WHO recommendation for the rational use of medications for outpatients in the developing countries, the optimal proportion of injection should be 13.4% - 24.1% of total prescriptions [3]. The proportion of prescriptions with injections in the THCs in Sichuan was 51.40%, far exceeding the WHO standard. Worse than that, the proportion of using multiple injections in one prescription was as high as 39.90% overall. Specifically, the proportion of prescriptions with four or more injections for treating pneumonia, COPD and hypertension was as high as 42.42%, 28.75% and 16.28% respectively. These results reveal that the overuse of injections, particularly the excessive unrestricted use of multiple injections, has become a big problem at the rural healthcare facilities in Sichuan. This overuse of injection phenomenon was also reported in other developing countries in recent years, such as in Cambodia, India and Pakistan [26-30]. Some scholars speculated that the abuse of injections was inextricably linked to the medical consumption concept of the consumers [26,31]. There is still a popular belief in China that injections are more convenient and effective than oral medications. Since few people know about the pernicious consequences of injection abuse, it is very common that patients demand injection treatments for the purpose of quick recovery from sickness. On the other hand, from the doctors’ perspective, injections are not only making their patients satisfied, but also producing more revenues for the health care facilities and themselves [31]. The Department of Health of provincial and local governments should make special efforts to enhance health education on the rational use of injections for the general population, and develop more targeted policy and program management for prescription practice at rural health care facilities. Public health education can promote general knowledge on the potential dangers of irrational use of medical injections, with a goal of changing the conventional consumer attitudes.

In summary, these results discussed above indicate that the irrational use of medications or polypharmacy is a major problem in the rural THCs in Sichuan. In China, health centers in rural areas in general have poorer resources than district or city health facilities, and are hence less likely to attract doctors with higher degrees or better experiences [1]. This evidence suggests some possible causes of irrational use of medications: (a) the rural medical institutions’ pursuit of maximizing economic profits, (b) the lack of medical skills and knowledge of rational medical prescriptions among rural doctors, (c) the lack of basic medical devices for diagnosis which leads to polypharmacy to cover ambiguous diagnoses, and (d) a lack of guidelines and effective policy for rational medical prescription practice in primary health care institutions. The median cost per encounter in THCs in Sichuan was 16.30 Yuan ($2.59) in 2008, significantly lower than that of the national average 24.84 Yuan ($3.94) in the same period [18]. This suggests that the irrational prescription practice in Sichuan is not pursuing economic profits, but the non-competency (lack of medical skills and knowledge) of doctors and/or ambiguous or uncertain clinical diagnosis due to lack of functional medical diagnosis equipment. According to the data from the Facilities Survey of the 4th NHSS of China (2008), in Sichuan Province, a large proportion of the health professionals in rural areas had relatively low education and low professional quality. Almost half (49.4%) of them had only technical secondary school education (which is equivalent to a high school) [32]. The average number of medical practitioners in the THCs was lower than the national average (4 vs. 6), and the average number of assistant medical practitioners in the THCs was also lower than the national average (3 vs. 4) [18,32]. Meanwhile, a considerable amount of the rural THCs in Sichuan still lacked basic functional diagnostic equipment. Only 24.1% of the THCs were equipped with automatic biochemical analyzers, and 27.6% of them had spectrophotometers [32].

Furthermore, we analyzed the median number of CPM and western medicine prescribed per encounter among the 30 sampled THCs. The results showed a more evenly distributed median number of CPM per encounter, compared to the uneven distribution of the median numbers of western medicine prescribed per encounter among the THCs. This means the prescription practice for western medicine was not administered in the same pattern among these THCs. Particularly, a greater proportion of surveyed THCs (73%, or 22 out of 30 THC) prescribed four or more western medications per encounter. Thus, we speculate that the irrational prescription practice may also be related to the lack of quality control on prescription practice in certain THCs.

These data suggest that apart from taking some measures to advance the administration system and the regulations for the rational use of medications, the provincial government must take some effective and practical actions at the same time, such as providing further education and training programs (especially on the rational use of medication) for the rural doctors, and by improving the hardware facilities of the rural THCs to enhance more accurate clinical diagnoses and to reduce the irrational medication use. In addition, some Chinese scholars have suggested establishing a public reporting system to supervise the prescription practices of the doctors [33].

This study also discovered a discrepancy between self-reported disease prevalence and medication prescription. Based on the data from the Household Health Survey of the 4th NHSS of China (2008), in Sichuan Province, the self-reported two-week prevalence of hypertension was 10.0 ‰ and the self-reported chronic hypertension was 28.0 ‰ among the rural residents, which were ranked at 6th place and 1st place of all the diseases, respectively, both ahead of self-reported prevalence of COPD [32]. Nevertheless, this study showed that the number of prescriptions for hypertension ranked only 10th, which was far behind that of COPD. The results of the Prescription Survey and the Household Health Survey appeared to be contradictory to each other. These conflicting results suggest that many hypertensive patients in rural areas did not seek treatment, and only a few patients made outpatient visits. The reasons for this phenomenon may be as follows: first, a lot of rural patients refuse to have medical treatment because of the ignorance of their health, or some of them may just be ignorant about the risk of hypertension; second, many patients may also choose self-medication instead of seeing a doctor to save money, because patients can buy most medications at a drugstore in China without a prescription from a doctor [34-36]. Therefore, health education on hypertension prevention and control in rural areas becomes more apparently important, especially with a focus on encouraging hypertensive patients to seek and comply with medical treatment. The THCs should strengthen the hypertension prevention and control program according to the Health Services Management Criteria for Hypertension (MoH of China, 2011) [37]. For example, local THCs should carry out a hypertension screening program yearly, and provide a face-to-face follow-up treatment for the patients with essential hypertension at least four times a year.

In conclusion, this study revealed certain issues of prescriptions practice in the THCs of Sichuan. Over-prescription of western medications (polypharmacy), antibiotics, injections, and irrational combination use of antibiotics and glucocorticoids are the major issues. The reasons for such irrational medication uses are not for maximizing profits of the THCs, but are more likely attributable to the lower competency of rural doctors, lack of medical devices for disease diagnosis in the THCs, lack of quality control policy and programs on prescription practice in the THCs, and lack of basic health knowledge on the adverse side effects of polypharmacy in general rural populations. Health policy on the quality control of prescription practice, professional development for rural doctors, and health education to rural residents could be essential to enhance prescription practice in the rural THCs and improve the health conditions of rural residents in Sichuan Province.

### Limitations

There are some limitations in our study. The study is descriptive without in-depth analysis of medications by name and dosage of specific medicines, and by physical condition of the patients. This is due to the fact that the Prescription Survey of the 4th NHSS did not collect such information. Furthermore, the study also could not provide more information and an in-depth discussion about the utilization of the Chinese patent medicine because of its complex chemical composition — a Chinese patent medicine usually contains several or even dozens of different components, in one word, it is a complex ‘gray system’ [38].

In the process of administering the Prescription Survey of the 4th NHSS, the MoH of China and provincial health bureaus took a series of measures to control the quality of the survey strictly. However, thousands of prescriptions were collected and only a small portion of questionnaires (5% of the total) were checked and reviewed. Therefore, misstatements or omissions, which we could not predict and avoid, might exist. Furthermore, we could not collect prescriptions through the whole year. This may affect the disease distribution observed in the study because of the limited survey period. All of these limitations may impact the results of the survey and our study.

## Competing interests

The authors declare that they have no competing interests.

## Authors’ contributions

QJ was the principal author of the manuscript. QJ, BY, JZ, HG, GY and JB contributed to the design of this study. QJ, JL and JZ performed data analysis. All authors contributed to the interpretation of the data, the drafting of manuscript, and have read and given approval to the final version of the manuscript.

## Pre-publication history

The pre-publication history for this paper can be accessed here:

http://www.biomedcentral.com/1472-6963/12/324/prepub
